# Interaction between Polyphenolic Antioxidants and *Saccharomyces cerevisiae* Cells Defective in Heavy Metal Transport across the Plasma Membrane

**DOI:** 10.3390/biom10111512

**Published:** 2020-11-04

**Authors:** Lavinia Liliana Ruta, Ileana Cornelia Farcasanu

**Affiliations:** Department of Organic Chemistry, Biochemistry and Catalysis, Faculty of Chemistry, University of Bucharest, Sos. Panduri 90–92, 050663 Bucharest, Romania; lavinia.ruta@chimie.unibuc.ro

**Keywords:** polyphenols, heavy metals, transport, *Saccharomyces cerevisiae*

## Abstract

Natural polyphenols are compounds with important biological implications which include antioxidant and metal-chelating characteristics relevant for their antimicrobial, antitumor, or antiaging potential. The mechanisms linking polyphenols and heavy metals in their concerted actions on cells are not completely elucidated. In this study, we used the model eukaryotic microorganism *Saccharomyces cerevisiae* to detect the action of widely prevalent natural polyphenols on yeast cells defective in the main components involved in essential heavy metal transport across the plasma membrane. We found that caffeic and gallic acids interfered with Zn accumulation, causing delays in cell growth that were alleviated by Zn supplementation. The flavones morin and quercetin interfered with both Mn and Zn accumulation, which resulted in growth improvement, but supplemental Mn and especially Zn turned the initially benefic action of morin and quercetin into potential toxicity. Our results imply that caution is needed when administering food supplements or nutraceuticals which contain both natural polyphenols and essential elements, especially zinc.

## 1. Introduction

Heavy metals are important environmental actors with a significant impact on biological systems [1]. Among them, the essential heavy metals (Cu, Co, Fe, Mn, Ni, Zn) are necessary in trace amounts for sustaining a number of biological functions, among which the role of enzymatic cofactors is by far the most important [2]. When present in high concentrations, heavy metals are toxic, as they damage the plasma membrane, bind nonspecifically to biomolecules, and interfere with the homeostasis of essential metals by competing with their normal transport and buffering systems [3,4]. A major route of heavy metal toxicity is determined by their capacity to generate oxidative stress, usually associated with radical production via Fenton-like reactions or with binding to chelating active groups of natural antioxidants, such as the thiol group in glutathione [5].

To meet the requirement of controlled low levels of essential metals, living organisms have developed intricate mechanisms of uptake, extrusion, and buffering. The buffering mechanism ensures the annihilation of the damaging effects of free metal ions by chelation, with one universal “ligand” against metal cytotoxicity being the group of cysteine-rich metal-binding proteins collectively known as metallothioneins [6]. The chelating strategy used by living organisms against metal toxicity has prompted the possibility to use chelation therapy for the treatment of heavy metal poisoning [4,7].

Among the natural compounds commonly present in the human diet, polyphenolic antioxidants have been considered as potential chelators of heavy metals [8,9], and there are several reports on plant antioxidants which alleviate Cd [10,11,12], Cu [13,14], Fe [15,16], and Mn [17,18] stresses. Dietary polyphenols are known not only for their antioxidant traits, but also for their protective roles in inflammation [19,20,21]; diabetes, obesity, and metabolic syndrome [22,23,24,25]; stroke prevention and cardiovascular health [26,27,28]; cancer prevention [29,30]; neuroprotection and antiaging [31,32]; sports performance [33,34]; etc. With so many health benefits it is no wonder that plant polyphenols have become important ingredients of food supplements and nutraceuticals available on the market. While the health benefits brought by the daily intake of natural polyphenols are undeniable, evidence suggests that consuming antioxidants in excess may not be entirely benefic, since at high concentrations many antioxidants can act as pro-oxidants, increasing the oxidative stress and inducing toxicity regarded as “antioxidative stress” [35,36,37].

To unravel new facets of natural polyphenols’ potential toxicity, we used *Saccharomyces cerevisiae* as a model for eukaryotic cells. With tractable genetics, exhaustively annotated genome, and ease of manipulation, *S. cerevisiae* has been extensively used in research studies designed to elucidate molecular mechanisms potentially extrapolatable to higher organisms [38,39]. *S. cerevisiae* has been previously used to study the effect of natural compounds on heavy metal toxicity [12,40,41]. Such studies predominantly involved exposure to metal excesses, but one study showed that curcumin, a polyphenol derived from turmeric, inhibited the growth of *S. cerevisiae* with defects in iron transport and homeostasis through iron chelation, which induced iron-depletion conditions incompatible with cell growth [42]. To determine whether other natural polyphenols interact with the homeostasis of essential heavy metals in yeast, we screened a number of well-known polyphenols with antioxidant properties against a set of knock-out *S. cerevisiae* mutants having individual deletions in the genes encoding plasma membrane transporters for heavy metals.

In *S. cerevisiae*, the transport of essential heavy metals across the plasma membrane is ensured by specific transporters with both high and low affinity [43]. These transporters include Ctr1 (Cu transporter [44]), Fet3/Ftr1 (complex involved in transport of Fe and Cu, [45]), Fet4 (low-affinity transporter for Fe and other transition metals [46]), Pho84 (phosphate transporter and a low-affinity divalent metal transporter [47,48]), Smf1 (divalent metal ion transporter with broad specificity and with high affinity for Mn [49]), Zrt1 (high-affinity Zn transporter [50]), Zrt2 (low-affinity Zn transporter [51]). The data obtained in this study unraveled new aspects which link the polyphenolic antioxidants to metal homeostasis in yeast cells and raise some questions regarding the safety of combining polyphenols and essential metals in food supplements and nutraceuticals.

## 2. Materials and Methods

### 2.1. Reagents and Growth Media

All reagents used, including yeast media components, were purchased from Merck (Darmstadt, Germany). Yeast strains were manipulated and maintained as described in [52] on yeast-peptone-dextrose (YPD, 1% *w*/*v* yeast extract, 2% *w*/*v* peptone, 2% *w*/*v* glucose) or synthetic complete media (SC, 0.67% *w*/*v* yeast nitrogen base with (NH_4_)_2_SO_4_, 2% *w*/*v* glucose, supplemented with the necessary amino acids). Minimal defined media (MM) were prepared adding individual components as described in [52] and contained 2 µM MnCl_2_, 2 µM ZnCl_2_, 1 µM FeCl_3_, and 0.1 µM CuCl_2_. For solid media, 2% *w*/*v* agar was used. The polyphenols were added to yeast media from 5 mM stock solutions prepared in dimethylsulfoxide (DMSO) and sterilized by filtration (0.22 μm pore size, Millipore, Billerica, MA, USA). The polyphenols used were caffeic acid (PubChem identity number, CID: 102261219), chlorogenic acid (CID: 1794427), cyanidin (CID: 128861), (−)-epicatechin (CID: 72276), epigallocatechin-3-*O*-gallate (CID: 65064), gallic acid (CID: 370), morin (CID: 5281670), quercetin (CID: 5280343), resveratrol (CID: 445154), and rutin (CID: 5280805).

### 2.2. Yeast Strains and Storage

The *S. cerevisiae* haploid strains used in this study were isogenic and had the wild-type (WT) background of BY4741 (*MAT*a; *his3Δ1*; *leu2Δ0*; *met15Δ0*; *ura3Δ0*). The knock-out strains had the genotype BY4741 *orf::kanMX4*, where the gene open reading frame (*ORF*) had been replaced by *kanMX4*. The strains were obtained from EUROSCARF [53] and are designated *orfΔ* throughout the manuscript. These strains were *ctr1Δ*, *ftr1Δ*, *fet4Δ*, *pho84Δ*, *smf1Δ*, *zrt1Δ*, and *zrt2Δ*.

### 2.3. Growth Assessment of the Yeast Strains

Wild type and *orfΔ* cells were inoculated from cells exponentially growing on YPD to MM liquid medium containing various polyphenols added from 5 mM DMSO stocks. Growth was monitored by measuring the turbidity of cell cultures at 600 nm (OD_600_) in a plate reader equipped with thermostat and shaker (Varioskan, Thermo Fisher Scientific, Vantaa, Finland).

### 2.4. Multielemental Analysis of Yeast Cells

Metal content of yeast cells was assessed as described in [54], with slight modifications. Cells exponentially growing on YPD were washed and suspended in MM liquid medium to OD_600_ = 0.1 in the absence or presence of individual polyphenols (100 μM, final concentration). The cells were incubated with shaking (200 rpm) for 16 h at 30 °C before they were harvested and washed three times with ice-cold 10 mM 2-(N-morpholino)ethanesulfonic acid (MES)–Tris buffer, pH 6.8. Cells were finally suspended in deionized water (10^8^ cells/mL) and used for both metal and cell protein assays. Metal content analysis was done using an instrument with a single collector, quadrupole inductively coupled plasma with mass spectrometry (ICP-MS, Perkin-Elmer ELAN DRC-e, Concord, Vaughan, ON, Canada) with axial field technology for trace elements, rare earth elements, and isotopic analyses. Metal analyses were performed after digestion of cells with 65% ultrapure HNO_3_ (Merck, Darmstadt, Germany). Standard solutions were prepared by diluting a 10 µg/mL multielement solution (Multielement ICP Calibration Standard 3, matrix 5% HNO_3_, PerkinElmer Pure Plus, Shelton, CT, USA). The cellular metal content was normalized to total cellular proteins, which were assayed spectrophotometrically [55].

### 2.5. Gene Expression Analysis by Quantitative Real Time-PCR (qRT-PCR)

Cells exponentially growing on YPD were washed and suspended in MM liquid medium to OD_600_ = 0.1 and grown to OD_600_ = 0.5 (approximately 6 h) before individual polyphenols were added (100 μM, final concentration). Cells were harvested after 1 h of polyphenol exposure, and total RNA was extracted using the RiboPure RNA Purification Kit for yeast (Ambion, Thermo Fischer Scientific, Vilnius, Lithuania). Approximately 500 ng RNA was transcribed into cDNA using GoScript Reverse Transcription System (Promega, Madison, WI, USA), and then 10 ng cDNA was used for each qRT-PCR done with the GoTaq qPCR Master Mix (Promega, Madison, WI, USA). Each reaction was performed in triplicate using MyiQ Single-Color Real-Time PCR Detection System (BioRad, Hercules, CA, USA). Expression of *ZAP1*, *ZRT1*, and *ZRT2* mRNA was normalized to the relative expression of *ACT1* in each sample, using the primers and PCR cycling conditions described in [54].

### 2.6. Reproducibility of the Results and Statistics

All experiments were repeated at least three times. For each individual measurement, values were expressed as the mean ± standard error of the mean (SEM). The data were examined by analysis of variance with multiple comparisons (ANOVA) using the statistical software Prism version 6.05 for Windows (GraphPad Software, La Jolla, CA, USA). One sample *t*-test was used for the statistical analysis of each strain/condition compared with a strain/condition considered as reference. The differences were considered to be significant when *p* < 0.05.

## 3. Results

### 3.1. Effect of Polyphenolic Antioxidants on the Growth of Yeast Cells Defective in Heavy Metal Transporters

Heavy metals are often related to oxidative stress, but most studies are done under metal excess conditions. Polyphenols are plant antioxidants that reportedly alleviate heavy metal stress induced by overexposure [10,11,12,13,14,15,16,17,18], although the chelating traits of polyphenols may result in essential metal deficit under normal conditions, such as was reported for iron [42]. To determine whether polyphenols affect the homeostasis of essential metals, we took a different approach and tested whether the exposure to natural polyphenolic antioxidants is compatible with the growth of *S. cerevisiae* cells bearing individual knock-out mutations in the genes encoding plasma membrane transporters for heavy metals. For this purpose, a number of widespread natural antioxidants were selected, namely (in alphabetical order) caffeic acid, chlorogenic acid, cyanidin, (−)-epicatechin, epigallocatechin-3-*O*-gallate, gallic acid, morin, quercetin, resveratrol and rutin. Each polyphenol was added to yeast cultures at 100 μM final concentration, which considerably surpassed the trace metal concentrations of the minimal defined medium (MM) used.

We tested the effect of polyphenols on the growth of cells lacking individual plasma membrane proteins involved in heavy metal transport: *ctr1Δ* (lacking the Ctr1 Cu transporter), *ftr1Δ* (lacking the Ftr1 Fe/Cu transporter), *fet4Δ* (lacking the Fet4 low-affinity transporter for Fe and other heavy metals), *pho84Δ* (lacking the Pho84 high-affinity phosphate transporter also involved in low-affinity transport heavy metals), *smf1Δ* (lacking the Smf1 high-affinity Mn transporter also involved in low-affinity transport of other heavy metals), *zrt1Δ* (lacking the Zrt1 high-affinity Zn transporter), and *zrt2Δ* (lacking the Zrt2 low-affinity Zn transporter). Since polyphenols are also known for their metal-chelating potential, we hypothesized that they may interfere with the growth of some of the mutants enumerated above. We, therefore, measured the growth of each strain in the presence of each polyphenol tested, considering that any growth alteration of a minimum of 25% (either up or down) would indicate a response of a strain to a specific polyphenol. None of the polyphenols used were toxic to the WT parental strain (Table 1). We also found that chlorogenic acid, cyanidin, (−)-epicatechin, epigallocatechin-3-*O*-gallate, resveratrol, and rutin did not significantly alter the growth of any of the strains used (Table 1). On the other side, caffeic and gallic acids inhibited cell growth of *zrt2Δ* cells by approximately 39% and 35%, respectively (Table 1), while morin and quercetin stimulated the cell growth of *fet4Δ*, *smf1Δ*, and *zrt1Δ* (Table 1). Therefore, we focused our subsequent experiments on the responsive polyphenols and strains.

### 3.2. Polyphenols Alter the Heavy Metal Content of Yeast Cells

Since some of the polyphenols affected the growth of yeast cells defective in heavy metal transport across the plasma membrane, we wondered whether this was the result of polyphenols interfering with metal accumulation. Therefore, we determined the cellular metal content of cells exposed to the selected polyphenols in media containing normal concentrations of essential heavy metals (Table 2).

It was noted that caffeic and gallic acids caused a significant decrease in the Zn content of *zrt2Δ* cells (Table 2), indicating a possible interaction of the two polyphenols with the Zn ions existent in the MM medium, an interaction which would affect the *zrt2Δ* growth (Table 1). Incubation with caffeic and gallic acids also caused a decrease in Zn content of WT and *zrt1Δ* cells (Table 2), but it was apparently not big enough to interfere with their growth (Table 1).

Morin and quercetin, on the other hand, did not interfere with normal Zn accumulation but instead caused significant drops in Mn content of *fet4Δ* and *smf1Δ* (Table 2) which were accompanied by the robust growth of these strains (Table 1). The *zrt1Δ* cells also showed better growth in the presence of both morin and quercetin (Table 1), but this was accompanied by low Zn accumulation rather than low Mn accumulation (Table 2).

### 3.3. Supplementary Zn Is Stimulative of Yeast Cell Growth in the Presence of Caffeic and Gallic Acids

**Figure 1:**
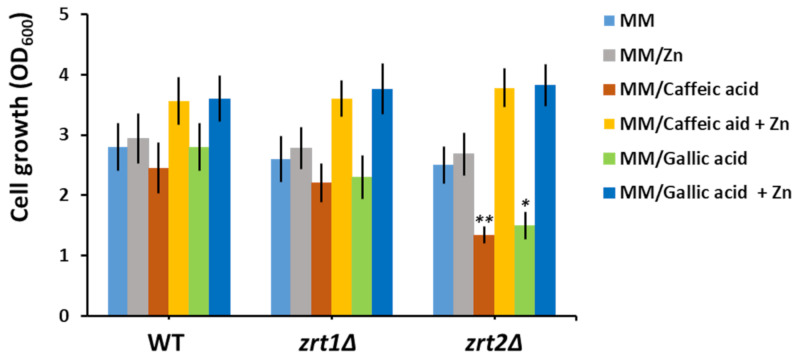
Effect of zinc supplementation on the growth of cells exposed to caffeic and gallic acids. Exponentially growing wild type (WT), *zrt1Δ*, and *zrt2Δ* cells were inoculated (OD_600_ = 0.05) in liquid minimal defined medium (MM) supplemented with the indicated compounds. Cell growth was determined spectrophotometrically (OD_600_) after 16 h of incubation (200 rpm, 30 °C). Values are mean ± SEM of triplicate determination done on three biological repeats. The concentrations of caffeic acid, gallic acid, and supplementary Zn were 100 µM each. Two-way ANOVA; * *p* < 0.05, ** *p* < 0.01.

It was intriguing to note that both caffeic and gallic acids preferentially caused a decrease in Zn accumulation by *zrt2Δ* cells compared to WT and *zrt1Δ* (Table 2). It was revealed that Zn deficiency indirectly correlates with increased oxidative stress caused by accumulation of reactive oxygen species (ROS) [56]; therefore, we wondered whether the increased sensitivity of *zrt2Δ* may be correlated to increased oxidative stress. Nevertheless, we could not find any significant difference between redox states of WT, *zrt1Δ* and *zrt2Δ*. We further tested whether the *zrt2Δ* sensitivity to caffeic and gallic acids may be alleviated by supplementary Zn. We found that adding Zn to the incubation media significantly improved the growth of not only *zrt2Δ* but also WT and *zrt1Δ* cells (Figure 1).

These results suggest that both caffeic and gallic acid may bind the Zn ions from the growth media, rendering them unavailable to *zrt2Δ* cells in a sufficient amount, thus affecting their growth rate. That supplementary Zn also improved the growth of wild type and *zrt1Δ* indicated that the caffeic or gallic acids competed for Zn with the yeast cells in all cases but caused serious Zn deprivation only in *zrt2Δ* cells (Table 2).

**Figure 2:**
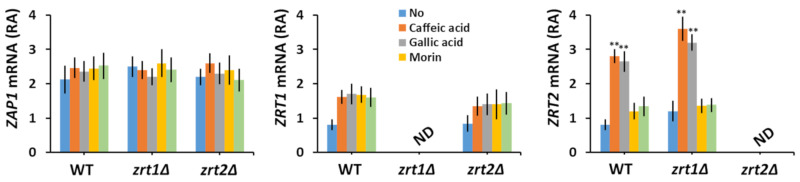
Effect of polyphenol exposure on the relative abundance (RA) of *ZAP1*, *ZRT1*, and *ZRT2* mRNA. Exponentially growing wild type (WT), *zrt1Δ*, and *zrt2Δ* cells were inoculated (OD_600_ = 0.1) in liquid minimal defined medium (MM) and grown 6 h before polyphenols were added (100 µM concentration). Cells were incubated for 1 additional hour before RNA isolation. Analysis of transcript abundance was done by qRT-PCR as described in Section 2. Expression of *ZAP1*, *ZRT1*, and *ZRT2* mRNA was normalized to the relative expression of *ACT1* in each sample. Values are mean ± SEM of triplicate qRT-PCR-s using cDNA obtained from three distinct colonies. Two-way ANOVA; ** *p* < 0.01. ND, not detected.

Zn uptake by *S. cerevisiae* cells is controlled at the transcriptional level by the Zn-responsive transcriptional activator Zap1, which regulates the transcription of *ZRT1* and *ZRT2* genes under Zn-limiting conditions [57], albeit in a different manner [58,59]. Therefore, we wondered whether polyphenol exposure may also modulate the expression of *ZRT1* or *ZRT2* genes. It was noticed that while not significantly changing the expression of *ZAP1* and *ZRT1*, caffeic and gallic acids stimulated *ZRT2* transcription (Figure 2).

### 3.4. Supplementary Mn and Zn Abrogate the Chemoprotective Effect of Morin and Quercetin

**Figure 3:**
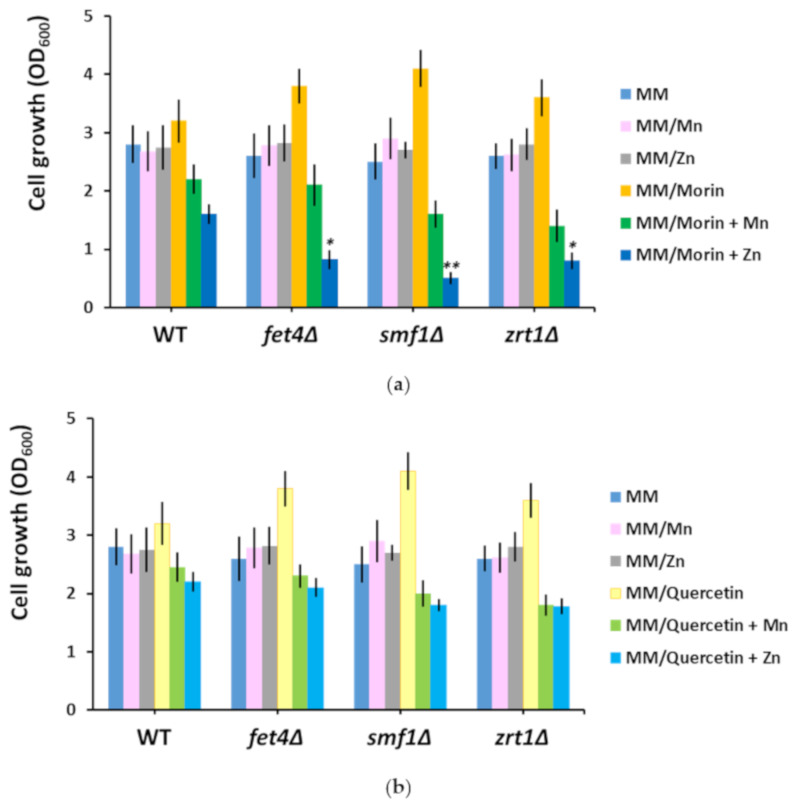
Effect of manganese and zinc supplementation on the growth of cells exposed to morin (**a**) and quercetin (**b**). Exponentially growing wild type (WT), *fet4Δ*, *smf1Δ*, and *zrt1Δ* cells were inoculated (OD_600_ = 0.05) in liquid minimal defined medium (MM) supplemented with the indicated compounds. Cell growth was determined spectrophotometrically (OD_600_) after 16 h of incubation (200 rpm, 30 °C). Values are mean ± SEM of triplicate determination done on three biological repeats. The concentrations of morin, quercetin, and supplementary Mn and Zn were 100 µM each. Two-way ANOVA; * *p* < 0.05, ** *p* < 0.01.

Both morin and quercetin, two flavonoids with metal chelating properties, improved the growth of fet4Δ and smf1Δ (Table 1), at the same time inducing a significant decrease in the Mn content of these mutants (Table 1). Another strain whose growth was improved by morin and quercetin was zrt1Δ, which showed reduced accumulation of Zn rather than Mn in the presence of the two flavones (Table 2). We subsequently determined the effect of supplementary Mn or Zn upon the growth of yeast cells exposed to morin or quercetin. It was noted that supplementary Mn reduced the growth of all three knock-out mutants in the presence of both morin (Figure 3a) and quercetin (Figure 3b). Supplementary Zn had a similar effect (Figure 3a,b), significantly affecting the growth of fet4Δ, smf1Δ, and zrt1Δ cells exposed to morin (Figure 3a). In fact, the Zn effect was rather deleterious; therefore, it can be easily speculated that supplementary Zn turns morin into a toxic compound.

## 4. Discussion

The association between polyphenols and essential heavy metals is an intensively studied topic of research, as both groups can have dual roles in modulating oxidative stress, acting as alleviators or enhancers, and it is not seldom that systems involved in oxidative stress are also involved in heavy metal homeostasis [60,61,62,63]. Besides their roles as both antioxidants (scavengers of oxidative reactive species) and pro-oxidants (generators of oxidative reactive species), polyphenols and heavy metals can also interact directly, thanks to the chelating properties of many polyphenols. In this study, we took advantage of the possible interaction between polyphenols and heavy metal ions to highlight new aspects fit to assess their potential toxic or beneficial roles. By screening a number of polyphenols against *S. cerevisiae* knock-out mutants defective in the transport of essential heavy metals across the plasma membrane, we found that caffeic and gallic acids were toxic to *zrt2Δ* cells, probably by reducing the availability of external Zn to *zrt2Δ* cells to an extent which affected cell growth. In this line of evidence, it was noted that caffeic and gallic acids increased the *ZRT2* transcription (Figure 2, right), probably by lowering the number of free ions in the cell environment. Interestingly, *ZRT1* mRNA abundance was not significantly altered by caffeic or gallic acid exposure (Figure 2, middle), indicating that neither of the two acids caused Zn depletion to a level that was low enough to induce *ZRT1* transcription. In support of this idea, it was reported that *ZRT1* and *ZRT2* transcription is differentially regulated depending on Zn availability: *ZRT1* under severe Zn depletion and *ZRT2* under “milder” depletion [57,58,59]. It was surprising to note that both caffeic and gallic acids were toxic to *zrt2Δ* but not to *zrt1Δ* (Table 1); since *zrt1Δ* cells had higher Zn content (Table 2) it became obvious that in the presence of caffeic and gallic acids Zn is preferentially taken up via Zrt2, which is expressed in WT and *zrt1Δ* but not in *zrt2Δ* cells. Remarkably, the polyphenols with caffeate and gallate moieties tested (i.e., chlorogenic acid and epigallocatechin-3-*O*-gallate, respectively) had no similar effect on *zrt2Δ* cells, most probably because their carboxylate group was esterified. Nevertheless, the interaction of nonresponsive polyphenols and heavy metals cannot be completely ruled out, but it may be too weak to be phenotypically detected under the experimental setup used in this study. In fact, epigallocatechin-3-*O*-gallate was shown to interfere with copper uptake by Fet3/Ftr1 by creating a local reductive environment [63].

It was interesting to observe the action of morin and quercetin on cells with defects in heavy metal uptake. Both polyphenols belong to the flavone class of antioxidants, with important metal-chelating characteristics [64]. Although isomers with similar structures, morin and quercetin have different pharmacokinetics [65]. While it was clear that morin and quercetin lowered the Mn content of *fet4Δ* and *smf1Δ* and the Zn content of *zrt1Δ*, it was not obvious why this reduction was paralleled by growth improvement. It is tempting to speculate that both morin and quercetin buffered the Mn or Zn to a concentration of free ions that is optimal for growth. Supplementing the media with otherwise nontoxic Mn or Zn turned the two flavones into villains which severely affected cell growth. This “breaking bad” turn was evident especially when morin was in combination with Zn rather than Mn (Figure 3a). Whether this behavior is the result of metal chelation is an issue still to be investigated. Both morin and quercetin are under scrutiny as potential antitumoral agents [66,67,68,69,70]; therefore, associating flavone with metal ion administration (especially Zn) may increase the anticancer potency of these drugs.

## 5. Conclusions

In this study it was found that caffeic and gallic acids interfered with Zn accumulation, causing delays in cell growth that were alleviated by Zn supplementation. The flavones morin and quercetin interfered with both Mn and Zn accumulation, which resulted in growth improvement, but supplemental Mn and especially Zn turned the initially benefic action of morin and quercetin into potential toxicity.

It was interesting to find that exposure of yeast cells to polyphenols resulted in altered Zn accumulation and that Zn supplementation could be both beneficial and detrimental to cells. Zn is one of the essential trace elements and one of the most encountered heavy metals in food supplements and nutraceuticals [71]. In light of our study, it is apparent that caution is needed when combining Zn supplementation with polyphenolic antioxidants and that further studies are needed to establish what plant antioxidants can be administered with Zn with no risk to human individuals.

## Figures and Tables

**Table 1:** Effect of polyphenolic antioxidants on the growth of various single-gene knockout mutants of *Saccharomyces cerevisiae*. Strains were inoculated (initial cell density 5 × 10^5^ cells/mL) in minimal defined media (MM) containing 100 μM polyphenol (final concentration). MM had controlled concentrations of trace elements, namely Cu^2+^ (0.1 μM), Fe^3+^ (1 μM), Mn^2+^ (2 μM), and Zn^2+^ (2 μM), which corresponded roughly to the concentrations of trace elements in rich media. Growth was assessed spectrophotometrically (OD_660_) after 16 h of incubation with shaking (200 rpm at 30 °C) and expressed as the ratio between cell densities in the presence and in the absence of the corresponding polyphenol. Results are mean ± standard error of the mean (SEM) of three independent determinations. The combination strain/polyphenol that determined improved growth by at least 25% (bold, *) or growth inhibition by at least 25% (bold, ^#^) were selected for further investigations.

Strain	Gene Deleted	Gene Function	Growth in the Presence of Antioxidant (%)	Antioxidant Tested
Wild Type ^1^	None	Control strain	0.94 ± 0.22	Caffeic acid
			0.88 ± 0.14	Chlorogenic acid
			0.96 ± 0.22	Cyanidin
			0.89 ± 0.25	(−)-Epicatechin
			1.12 ± 0.21	Epigallocatechin-3-*O*-gallate
			0.91 ± 0.24	Gallic acid
			1.24 ± 0.14	Morin
			1.28 ± 0.26	Quercetin
			0.95 ± 0.14	Resveratrol
			0.96 ± 0.23	Rutin
**Genes Involved in Heavy Metal Transport**				
*ctr1Δ*	*CTR1*	High-affinity copper transporter of plasma membrane	0.92 ± 0.24	Caffeic acid
			0.89 ± 0.12	Chlorogenic acid
			0.93 ± 0.21	Cyanidin
			0.91 ± 0.18	(−)-Epicatechin
			0.88 ± 0.17	Epigallocatechin-3-*O*-gallate
			0.93 ± 0.22	Gallic acid
			0.96 ± 0.14	Morin
			1.08 ± 0.16	Quercetin
			0.91 ± 0.14	Resveratrol
			0.90 ± 0.17	Rutin
*ftr1Δ*	*FTR1*	High-affinity iron transporter of the plasma membrane	0.90 ± 0.17	Caffeic acid
			0.91 ± 0.14	Chlorogenic acid
			0.93 ± 0.16	Cyanidin
			0.92 ± 0.15	(−)-Epicatechin
			0.78 ± 0.34	Epigallocatechin-3-*O*-gallate
			0.84 ± 0.26	Gallic acid
			0.84 ± 0.25	Morin
			0.88 ± 0.28	Quercetin
			0.91 ± 0.16	Resveratrol
			0.93 ± 0.24	Rutin
*fet4Δ*	*FET41*	Low-affinity iron transporter of the plasma membrane	0.92 ± 0.23	Caffeic acid
			0.98 ± 0.17	Chlorogenic acid
			0.94 ± 0.16	Cyanidin
			0.92 ± 0.15	(−)-Epicatechin
			0.91 ± 0.21	Epigallocatechin-3-*O*-gallate
			0.90 ± 0.28	Gallic acid
			**1.43 ± 0.16** *	**Morin** *
			**1.45 ± 0.25** *	**Quercetin** *
			0.98 ± 0.24	Resveratrol
			0.94 ± 0.22	Rutin
*pho84Δ*	*PHO84*	High-affinity inorganic phosphate (Pi) transporter and low-affinity divalent metal ion transporter	0.80 ± 0.23	Caffeic acid
			0.78 ± 0.14	Chlorogenic acid
			0.76 ± 0.22	Cyanidin
			0.79 ± 0.25	(−)-Epicatechin
			0.82 ± 0.21	Epigallocatechin-3-*O*-gallate
			0.85 ± 0.24	Gallic acid
			0.78 ± 0.21	Morin
			0.79 ± 0.25	Quercetin
			0.85 ± 0.14	Resveratrol
			0.84 ± 0.21	Rutin
*smf1Δ*	*SMF1*	Divalent metal ion transporter	0.92 ± 0.25	Caffeic acid
			0.94 ± 0.14	Chlorogenic acid
			1.11 ± 0.21	Cyanidin
			0.93 ± 0.15	(−)-Epicatechin
			1.02 ± 0.20	Epigallocatechin-3-*O*-gallate
			0.91 ± 0.24	Gallic acid
			**1.56 ± 0.34** *	**Morin** *
			**1.47 ± 0.23** *	**Quercetin** *
			0.98 ± 0.24	Resveratrol
			0.95 ± 0.21	Rutin
*ztr1Δ*	*ZTR1*	High-affinity Zn^2+^ transporter of the plasma membrane	0.94 ± 0.22	Caffeic acid
			0.88 ± 0.14	Chlorogenic acid
			0.96 ± 0.22	Cyanidin
			0.89 ± 0.25	(−)-Epicatechin
			1.12 ±0.21	Epigallocatechin-3-*O*-gallate
			0.91 ± 0.24	Gallic acid
			**1.48 ± 0.22** *	**Morin** *
			**1.46 ± 0.16** *	**Quercetin** *
			0.95 ± 0.14	Resveratrol
			0.96 ± 0.23	Rutin
*ztr2Δ*	*ZTR2*	Low-affinity Zn^2+^ transporter of the plasma membrane	**0.61 ± 0.14** ^#^	**Caffeic acid** ^#^
			0.88 ± 0.14	Chlorogenic acid
			0.96 ± 0.22	Cyanidin
			0.91 ± 0.15	(−)-Epicatechin
			0.92 ± 0.21	Epigallocatechin-3-*O*-gallate
			**0.65 ± 0.24** ^#^	**Gallic acid** ^#^
			1.24 ± 0.14	Morin
			1.15 ± 0.26	Quercetin
			0.95 ± 0.14	Resveratrol
			0.96 ± 0.23	Rutin

^1^ Wild type is strain BY4741 (*MAT***a**; *his3Δ1*; *leu2Δ0*; *met15Δ0*; *ura3Δ0*). The isogenic knock-out mutants had the phenotype BY4741 except for a null mutation of one single gene: *orf::kanMX4*. All strains were purchased from EUROSCARF [53].

**Table 2:** Effect of polyphenols on metal content of yeast cells. Exponentially growing cells were washed and shifted to MM (OD_600_ = 0.1) in the absence or presence of the antioxidants (100 μM each, final concentration). Cells were grown with agitation (30 °C, 200 rpm) for 16 h before being harvested for multielemental analysis. Each determination was done in triplicate on approximately 10^8^ cells from three biological replicates. Results are given as mean ± SEM (standard error of the mean). The level of significance was determined by one sample *t*-test, when each strain was compared with WT under the same conditions (hash sign, ^#^) or with the same strain grown without polyphenol (asterisk, *). ^#^ *p* < 0.05; ^##^ *p* < 0.01; * *p* < 0.05; ** *p* < 0.01.

Strain	Antioxidant	Cellular Metal Content (nmol/mg Cell Total Protein)			
		Cu	Fe	Mn	Zn
WT	None	5.84 ± 0.42	52.82 ± 3.24	6.25 ± 0.62	12.42 ± 1.14
	Caffeic acid	5.21 ± 0.31	50.78 ± 5.82	5.96 ± 0.38	8.48 ± 1.04
	Gallic acid	4.98 ± 0.52	49.34 ± 4.21	5.28 ± 0.51	9.25 ± 0.98
	Morin	4.82 ± 0.42	48.38 ± 4.54	4.21 ± 0.28	11.48 ± 1.16
	Quercetin	4.65 ± 0.63	47.22 ± 3.98	4.10 ± 0.31	11.22 ± 1.28
*fet4Δ*	None	5.24 ± 0.35	51.34 ± 2.81	4.25 ± 0.57 ^#^	11.48 ± 1.23
	Caffeic acid	5.04 ± 0.24	49.33 ± 3.62	4.15 ± 0.27	10.32 ± 1.11
	Gallic acid	4.52 ± 0.38	48.68 ± 3.29	4.28 ± 0.42	9.47 ± 0.87
	Morin	4.32 ± 0.33	48.02 ± 3.65	2.21 ± 0.36 ^#,^*	9.84 ± 0.92
	Quercetin	4.14 ± 0.41	47.82 ± 3.62	2.11 ± 0.31 ^#,^*	9.68 ± 1.08
*smf1Δ*	None	4.34 ± 0.42	50.82 ± 4.15	3.16 ± 0.51 ^#^	9.42 ± 1.21
	Caffeic acid	4.02 ± 0.34	50.31 ± 3.87	3,37 ± 0.39 ^#^	8.72 ± 1.05
	Gallic acid	4.23 ± 0.38	48.34 ± 4.49	3.23 ± 0.47 ^#^	8.25 ± 0.91
	Morin	4.56 ± 0.45	46.65 ± 4.39	1.21 ± 0.18 ^##,^*	8.43 ± 1.11
	Quercetin	4.37 ± 0.45	45.92 ± 3.77	1.10 ± 0.31 ^##,^*	8.22 ± 1.07
*zrt1Δ*	None	5.92 ± 0.53	53.92 ± 4.94	5.32 ± 0.42	8.68 ± 1.04 ^#^
	Caffeic acid	4.29 ± 0.38	52.75 ± 4.83	4.91 ± 0.37	7.37 ± 0.95 ^#^
	Gallic acid	4.56 ± 0.45	50.68 ± 5.21	4.78 ± 0.51	7.25 ± 0.92 ^#^
	Morin	4.82 ± 0.42	50.08 ± 4.59	4.57 ± 0.45	6.38 ± 0.56 ^#,^*
	Quercetin	4.68 ± 0.63	49.97 ± 4.98	4.71 ± 0.43	6.22 ± 0.73 ^#,^*
*zrt2Δ*	None	4.62 ± 0.51	54.31 ± 4.14	5.72 ± 0.84	10.61 ± 0.89
	Caffeic acid	4.22 ± 0.44	55.25 ± 4.32	5.38 ± 0.65	3.47 ± 1.02 ^##,^**
	Gallic acid	4.17 ± 0.42	51.44 ± 6.12	5.09 ± 0.61	4.25 ± 0.93 ^##,^**
	Morin	4.22 ± 0.40	50.25 ± 5.24	2.98 ± 0.28 *	9.26 ± 1.05
	Quercetin	4.12 ± 0.51	50.86 ± 4.63	2.34 ± 0.22 *	9.12 ± 1.28
